# Early Mortality and Primary Causes of Death in Mothers of Children with Intellectual Disability or Autism Spectrum Disorder: A Retrospective Cohort Study

**DOI:** 10.1371/journal.pone.0113430

**Published:** 2014-12-23

**Authors:** Jenny Fairthorne, Geoff Hammond, Jenny Bourke, Peter Jacoby, Helen Leonard

**Affiliations:** 1 Telethon Kids Institute, University of Western Australia, Perth, Australia; 2 Health Department of Western Australia, Perth, Australia; Hamamatsu University School of Medicine, Japan

## Abstract

**Introduction:**

Mothers of children with intellectual disability or autism spectrum disorder (ASD) have poorer health than other mothers. Yet no research has explored whether this poorer health is reflected in mortality rates or whether certain causes of death are more likely. We aimed to calculate the hazard ratios for death and for the primary causes of death in mothers of children with intellectual disability or ASD compared to other mothers.

**Methods:**

The study population comprised all mothers of live-born children in Western Australia from 1983–2005. We accessed state-wide databases which enabled us to link socio-demographic details, birth dates, diagnoses of intellectual disability or ASD in the children and dates and causes of death for all mothers who had died prior to 2011. Using Cox Regression with death by any cause and death by each of the three primary causes as the event of interest, we calculated hazard ratios for death for mothers of children intellectual disability or ASD compared to other mothers.

**Results and Discussion:**

During the study period, mothers of children with intellectual disability or ASD had more than twice the risk of death. Mothers of children with intellectual disability were 40% more likely to die of cancer; 150% more likely to die of cardiovascular disease and nearly 200% more likely to die from misadventure than other mothers. Due to small numbers, only hazard ratios for cancer were calculated for mothers of children with ASD. These mothers were about 50% more likely to die from cancer than other mothers. Possible causes and implications of our results are discussed.

**Conclusion:**

Similar studies, pooling data from registries elsewhere, would improve our understanding of factors increasing the mortality of mothers of children with intellectual disability or ASD. This would allow the implementation of informed services and interventions to improve these mothers' longevity.

## Introduction

Poorer health, particularly mental health, has consistently been documented in the mothers of children with intellectual disability [1], [2] or autism spectrum disorder (ASD). [3]–[5] To some extent, the degree of health impairment in mothers has been shown to vary according to the type of their child's disability. For example, in studies which compared the mental health of mothers of children with different developmental disabilities, mothers of children with Down syndrome had the least impaired health and mothers of those with ASD, the most impaired. [6]–[8] However whether or not the ASD is associated with comorbid intellectual disability, occurring in about 50% cases, may also have an impact. Moreover due to the increased independence of their children, mothers of children with mild intellectual disability are likely to have different challenges than mothers of children with severe intellectual disability.

Our previous research identified that mothers with a previous psychiatric disorder were about twice as likely to have a child with ASD than mothers without a psychiatric disorder [9] and we know that people with psychiatric disorders have higher mortality rates. [10] Hence, we were also interested in investigating whether the existence of a psychiatric disorder, either before or after the birth of the child, affected mortality. Diagnosis of ASD is increasing. [11] This, combined with the progressive closing of residential facilities for people with disabilities in developed nations, [12] has resulted in many more mothers now caring at home for their children with intellectual disability or ASD.

The examination of mortality rates and identifying any causes of early death which are more common in particular groups of mothers of children with intellectual disability or ASD would enable services and interventions to be directed to those whose health is most vulnerable. Thus, any increased mortality might be reduced, along with the corresponding emotional and financial burden to affected families and economic burden to the community.

Therefore, for the study period, we aimed to:

1. Estimate the survival rates in mothers of children with intellectual disability, ASD and in those whose children have neither intellectual disability or ASD
2. Compare the risk of death in mothers of children with different subgroups of intellectual disability and ASD compared to other mothers
3. Examine the extent to which a psychiatric disorder can explain any observed differences in mortality rates
4. Identify the primary causes of death in mothers of children with intellectual disability or ASD and estimate how the risks compare with mothers of children without these disabilities.

We achieved each of the above objectives.

## Methods

### Study population

The study population comprised all women who gave birth to a live child in Western Australia (WA) in the years from 1983 to 2005. Our de-identified data were obtained from five state-wide sources. The first was the *Midwives Notification System* (MNS) and from here we obtained mothers' socio-economic status (SES) and the birth dates of mothers and their babies. Secondly, children's diagnostic information, pertaining to the presence of intellectual disability (including type and level) or ASD (including whether associated with intellectual disability) was provided by the *Intellectual Disability Exploring Answers* (IDEA) Database. [13] Thirdly, from the state mortality registry, we accessed dates and cause of death by ICD-9 or 10 codes of all mothers in the study population who had died from 1983 to 2010. Lastly, in order to explore the effect of a psychiatric disorder on mortality, we accessed data-sets from the Mental Health Information Service (MHIS) and the Hospital Morbidity Data System (HMDS). We linked all data-sets by using a unique alpha-numeric identifier created for each mother by WA's *Data Linkage Unit*. [14]

### Maternal groups

**Figure 1:**
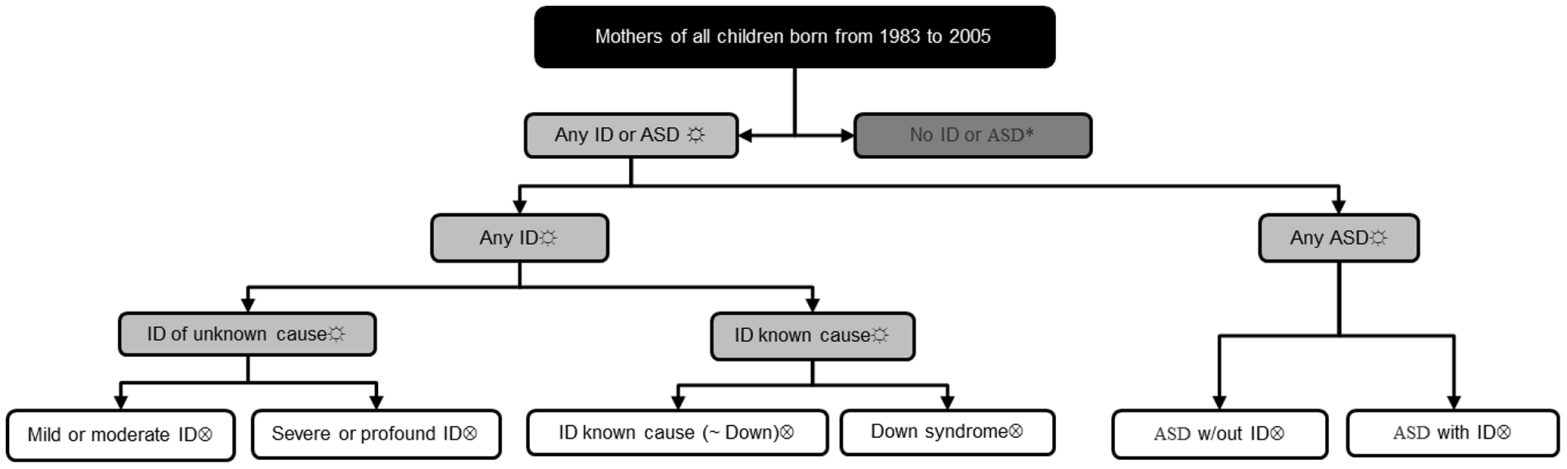
Comparison group, composite and core case groups.

Initially, we formed the six core case groups of ‘mild intellectual disability of unknown cause’, ‘severe intellectual disability of unknown cause,’ Down syndrome, ‘intellectual disability of known cause (not Down syndrome)’, ASD with intellectual disability and ASD without intellectual disability. All children who had ASD with intellectual disability were considered only as an ASD case group and not as an intellectual disability case group. These were termed ‘core case groups’. We chose these groups carefully as inappropriate grouping would mask differential mortality within a sub-group. For example, it was important to separate the mothers of children with Down syndrome from the mothers of children with other intellectual disability of known cause. Firstly, this was because Down syndrome is the most common known cause of intellectual disability. Secondly, not being an inherited disorder, mothers would not have any related, genetically determined effects on their health as might occur with other conditions such as Fragile X or neurofibromatosis. Thirdly, the mothers of children with Down syndrome have not experienced a particular exposure (such as heavy alcohol consumption or severe anaemia) which might be associated with their child's disability and which might also impact their mortality. The comparison group consisted of all mothers with no child with intellectual disability or ASD. Next, we combined selected core case groups to form two composite case groups, mothers of children with intellectual disability (excluding those with ASD and comorbid intellectual disability), and mothers of children with ASD (including those with ASD and comorbid intellectual disability). We formed a third group, mothers of children with either intellectual disability or ASD, by combining the two composite case groups. These provided larger numbers for analyses as required. Each mother was assigned an index child and in mothers of children with intellectual disability or ASD, the index child was their eldest child with a disability born from 1983 to 2005. In the comparison group, the index child was the eldest child born from 1983 to 2005. Mothers were assigned to a case group according to the disability of their index child. In Fig. 1, the comparison, composite and core case groups and their inter-relationships are shown.

### Analyses

Using Kaplan-Meier analysis, [15] we calculated the survival rates of mothers of children with any intellectual disability, any ASD and no intellectual disability or ASD. All survival curves were tested for differences using the log-rank test for equality of survivor functions.

We tested the *Proportional Hazards Assumption* [16] with the dependent variable for the six core case groups and where failure was ‘Death by any cause’ and each of the primary cause of death categories. No hazard curve varied significantly from the base-line curve with respect to proportionality. Hence, we concluded that our data were suitable for Cox Regression analysis.

Maternal age and SES are related to both the risk of intellectual disability, [17] ASD [17] and death [18] and hence we adjusted for these potential confounders. We created a three level variable for age at the index birth: ‘Less than 20 years’, ‘20 to 35 years’ and ‘35 years or more’. Socio-economic status was measured by a three-level variable calculated from the *Indices of Relative Socioeconomic Disadvantage* [19] for 2001 which uses residence grouped by the unit termed ‘collection district’. For mothers where this was not available, we used the same index but with measures from 1996 or 2006 or a similar index for 2001 which used ‘Statistical local areas’ although larger than collection districts. In our variable, ‘low’ pertained to the most disadvantaged quartile of scores, ‘medium’ to the inner two quartiles and ‘high’ to the most advantaged quartile where we determined the quartiles from our study population.

Using Cox regression with ‘Death by any cause’ as the event of interest and time measured as the number of years since the index birth, we calculated the hazard ratios for death by maternal case group compared to the comparison group. The period at risk extended from the date of the index birth until death or 31^st^ December, 2010, whichever came first. We calculated unadjusted hazard ratios and hazard ratios adjusted for each and both of maternal age at the index birth and SES.

### Effect of a psychiatric disorder

We created a binary variable with the level of ‘Yes’ for women who had had an outpatient clinical psychiatric contact or a hospital admission in relation to a psychiatric diagnosis in WA during their life-time. Using the Kaplan-Meier method, we compared the survival rates in mothers according to the existence of both a psychiatric disorder and the disability status of the index child. We also calculated the hazard ratios for ‘Death by any cause’ for each of the three case groups and compared to mothers of children with no intellectual disability or ASD and no psychiatric disorder.

### Cause of death

We grouped the causes of death into the twelve categories of Infections or parasites, Cancer, Diabetes, Cardiovascular diseases, Respiratory diseases, pneumonia and influenza, Digestive diseases, Kidney diseases, Pregnancy complications, Misadventure, Other causes, Genetic and congenital disorders and Mental disorders. The first ten of these were formed from the 38 groups of the ICD-10 Cause of death codes. [20] The remaining two, Genetic and congenital disorders and Mental disorders were added as death was associated with these disorders for 59 mothers. The ICD codes pertaining to each diagnostic category are in S1 Table. We performed Cox Regression analyses where ‘failure’ was death attributed to each of the three largest causes of death categories. In turn, we used each of the three largest case groups: mothers of children with intellectual disability; mothers of children with intellectual disability and/or ASD and mothers of children with ASD as independent variables. Again, the period at risk extended until death from any cause or the end of the study period, whichever occurred first. The base level was the comparison group and we adjusted for maternal age at index birth and SES. STATA 12 was used for all analyses.

### Ethics statement

This study was approved by the Western Australian Department of Health Human Research Ethics Committee (#2011/64). Informed consent was not required from participants as all data were de-identified prior to the commencement of analysis.

## Results

### Study population

The original data-set contained the records of 300,123 mothers and of these 3,693 (1.2%) had died before 2011. Their mean age of death was 42 years and their ages at death ranged from 16 to 74 years. The maternal age at the index birth and SES of these women are given by maternal groups in Table 1.

**Table 1:** Demographic and psychiatric characteristics of the study population by number and percentage of maternal group.

Characteristic	No ID or ASD (Comparison group)	Mild ID (unknown cause)	Severe ID (unknown cause)	Down syndrome	ID of known cause (not Down)	Any ID	ASD with ID	ASD without ID	Any ASD N =	Any ID or ASD	Total
MATERNAL AGE AT THE INDEX BIRTH											
***Less than 20 years***	N = 22,042 7.6%	N = 607 10.7%	N = 26 7.1%	N = 17 2.9%	N = 89 7.9%	N = 739 9.6%	N = 52 3.7%	N = 28 4.4%	N = 80 3.9%	N = 819 8.4%	**N = 22,861 7.6%**
***20 - 34 years***	N = 235,644 81.2%	N = 4,476 79.0%	N = 283 77.7%	N = 365 62.7%	N = 869 77.5%	N = 5,993 77.5%	N = 1,080 76.7%	N = 494 78.0%	N = 1,574 77.1%	N = 7,567 77.4%	**N = 243,211 81.0%**
***35 years or more***	N = 32,662 11.2%	N = 584 10.3%	N = 55 15.1%	N = 200 34.4%	N = 163 14.5%	N = 1,002 13.0%	N = 276 19.6%	N = 111 17.5%	N = 387 19.0%	N = 1,389 14.2%	**N = 34,051 11.3%**
SOCIO-ECONOMIC STATUS											
***Low***	N = 64,570 22.2%	N = 2,297 42.3%	N = 129 35.4%	N = 115 19.8%	N = 374 33.4%	N = 2,915 37.7%	N = 351 24.9%	N = 144 22.8%	N = 495 24.3%	N = 3.410 34.9%	**N = 68,080 22.7%**
***Medium***	N = 144,610 49.8%	N = 2,466 43.5%	N = 167 45.9%	N = 305 52.4%	N = 522 46.6%	N = 3,460 44.7%	N = 706 50.1%	N = 320 50.6%	N = 1,026 50.3%	N = 4,486 45.9%	**N = 149,096 49.7%**
***High***	N = 72,380 24.9%	N = 687 12.1%	N = 56 15.4%	N = 156 25.1%	N = 319 22.7%	N = 1,218 15.7%	N = 319 22.7%	N = 150 23.7%	N = 469 23.0%	N = 1,687 17.3%	**N = 73,936 21.4%**
***Missing***	8.788 3.0%	117 2.1%	12 3.3%	27 2.4%	32 2.3%	188 2.4%	32 2.3%	N = 19 3.0%	N = 51 2.5%	N = 239 2.4%	**N = 9.011 3.0%**
EXISTENCE OF A PSYCHIATRIC DISORDER											
***Yes***	N = 50,585 17.4%	N = 2,018 35.6%	N = 113 31.0%	N = 120 20.6%	N = 387 34.5%	N = 2,638 34.1%	N = 365 25.9%	N = 184 29.1%	N = 549 26.9%	N = 3,187 32.6%	**N = 53,772 17.9%**
**TOTAL**	**290,348**	**5,667**	**364**	**582**	**1,121**	**7,734**	**1,408**	**633**	**2,041**	**9,775**	**300,123**

ID, intellectual disability; ASD, autism spectrum disorder; Mild ID, Mild or moderate intellectual disability; Severe ID, Severe or profound intellectual disability; Down, Down syndrome.

### Survival rates and death by any cause

**Figure 2:**
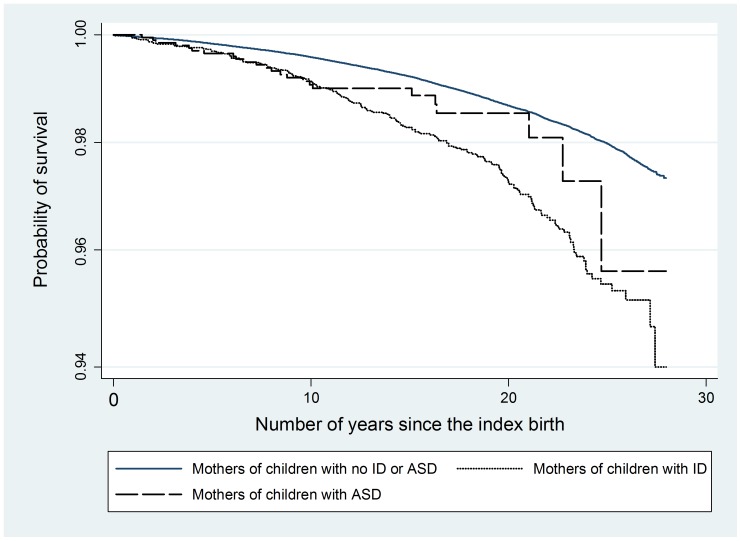
Kaplan-Meier survival rates of mothers of children with no intellectual disability or ASD, mothers of children with intellectual disability and mothers of children with ASD.

Twenty-five years after the birth of their index child, the survival rates of mothers of children with no intellectual disability and no ASD were about 98%, followed by 96% for mothers of children with ASD and 95% for mothers of children with intellectual disability. Mothers in both the intellectual disability (log-rank p-value  = 0.00005) and ASD (log-rank p-value  = 0.0436) case groups had significantly poorer survival than comparison mothers. The survival curves are in Fig. 2.

**Figure 3:**
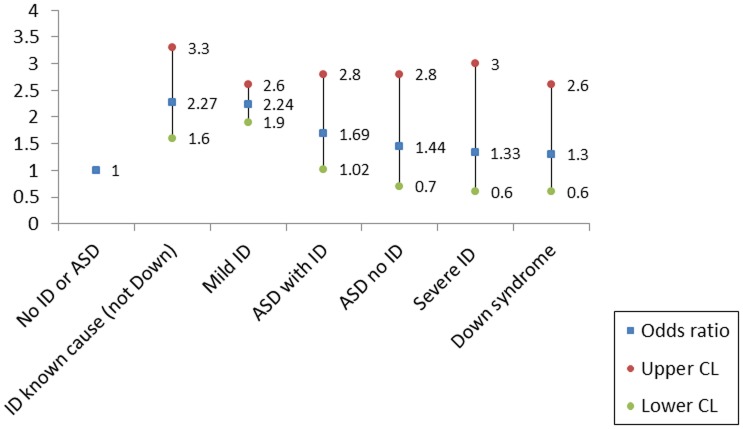
Adjusted* hazard ratios for death by maternal case group.

Before adjustment, all core case groups had greater risk of death by any cause. During the study period, mothers of children with *intellectual disability of known cause (not Down syndrome)* [2.31(95% CI: 1.6, 3.3)] and mothers of children with mild intellectual disability [2.29(95% CI: 1.9, 2.7)] had the highest risk of death by any cause. Mothers of children with Down syndrome [1.36(95% CI: 0.7, 2.7)] and mothers of children with severe intellectual disability [1.31(95% CI: 0.6, 2.9)] had the lowest risk of case mothers. In the final model, we adjusted for both maternal age at the index birth and SES. All hazard ratios were slightly attenuated and remained greater than one. Hazard ratios for the mothers of children with *intellectual disability of known cause (not Down syndrome)* [2.27(95% CI: 1.6, 3.3)], mothers of children with mild intellectual disability [2.24(95% CI: 1.9, 2.6)], and mothers of children with ASD and intellectual disability [1.71(95% CI: 1.02, 2.8)] were significant. (Fig. 3)

### Effect of a psychiatric disorder

**Figure 4:**
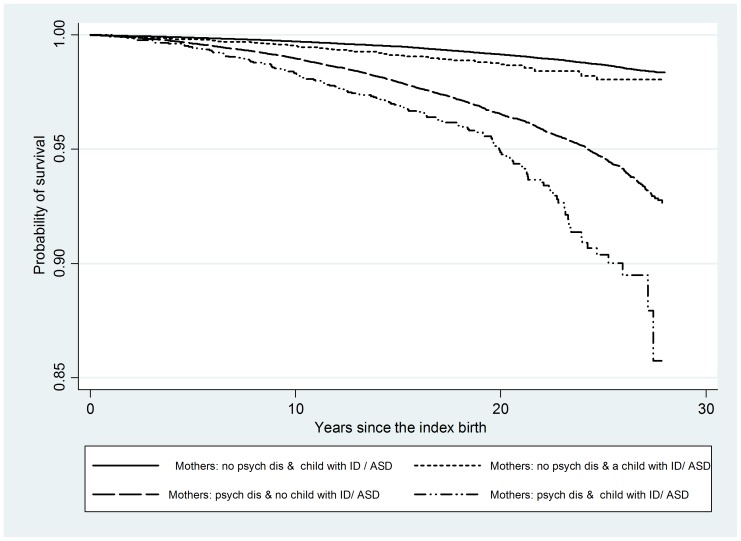
Kaplan-Meier survival rates of mothers according to the existence of a psychiatric disorder and the disability status of the index child.

We compared the survival of mothers with and without a psychiatric disorder and according to the disability status of their index child. Twenty-five years after the index birth, the survival rates of the comparison group and three case groups, in descending order, were as follows. Mothers with no psychiatric disorder and no child with intellectual disability or ASD had the highest survival rate of about 98.5%. Mothers with no psychiatric disorder and a child with intellectual disability had a 98% chance of survival. Mothers with a psychiatric disorder and no child with intellectual disability had a 95% chance of survival while mothers with a psychiatric disorder and a child with intellectual disability or ASD had a survival rate of about 90% (all associated p-values <0.00005). See Fig. 4. Mothers with both a psychiatric disorder and a child with intellectual disability or ASD had about six and a half times the risk of death and those with a psychiatric disorder and no child with intellectual disability or ASD had about four times the risk of death. Mothers with no psychiatric disorder but a child with intellectual disability or ASD had a 52% increased risk of death.

### Cause of death

The three primary causes of death in our cohort were cancer (N = 1,452), misadventure (N = 843) and cardiovascular diseases (N = 433). The ICD-9 and 10 codes for each of the ‘cause of death’ categories are in S1 Table. Elevated hazard ratios for death due to cancer ranged from 1.41(95% CI: 1.1, 1.8) for mothers of children with intellectual disability, and 1.54(95% CI: 0.8, 2.9) for mothers of children with ASD. The hazard ratios for dying of a cardio-vascular disease was 2.6(95% CI: 1.8, 3.9) in mothers of children with intellectual disability and 2.52(95% CI: 1.7, 3.7) in mothers of children with intellectual disability or ASD. For death by misadventure, hazard ratios were significantly increased for mothers of children with intellectual disability or ASD [1.75(95% CI: 1.3, 2.4)] and intellectual disability [1.95(95% CI: 1.4, 2.7)]. All hazard ratios and numbers of mothers by the case groups of intellectual disability, ASD and intellectual disability or ASD are shown in Table 2. Analyses were not performed in cells with numbers of five or less as results would have been unreliable. [15]

**Table 2:** Mothers experiencing death by case group and cause with hazard ratios and 95% confidence intervals for primary causes of death.

	Comparison group				
Category	No intellectual disability or ASD	Any intellectual disability	Any ASD	Any intellectual disability or ASD	Total
***Infections or parasites***	N = 46	N = 2	N = 0	N = 2	**50**
***Cancer***	N = 1,587	1.41 (1.1, 1.8) N = 55	1.54 (0.8, 2.9) N = 10	1.42 (1.1, 1.8) N = 65	**1,652**
***Diabetes***	N = 59	N = 6	N = 0	N = 6	**65**
***Cardiovascular diseases***	N = 404	2.6 (1.8, 3.9) N = 26	N = 3	2.52 (1.7, 3.7) N = 29	**433**
***Respiratory diseases, pneumonia and influenza***	N = 109	N = 6	N = 1	N = 7	**116**
***Digestive diseases***	N = 121	N = 16	N = 1	N = 17	**138**
***Kidney diseases***	N = 14	N = 6	N = 0	N = 6	**20**
***Pregnancy complications***	N = 17	N = 1	N = 1	N = 2	**19**
***Misadventure***	N = 797	1.95 (1.4, 2.7) N = 43	N = 3	1.75 (1.3, 2.4) N = 46	**843**
***Genetic and congenital disorders***	N = 18	N = 1	N = 2	N = 3	**21**
***Mental disorders***	N = 34	N = 4	N = 0	N = 4	**38**
***Other causes***	N = 273	N = 24	N = 3	N = 27	**300**
***Total***	**N = 3,479**	**N = 190**	**N = 24**	**N = 214**	**3,693**

ASD, autism spectrum disorder; Mild intellectual disability, Mild or moderate intellectual disability of unknown cause; Severe intellectual disability, Severe or profound intellectual disability of unknown cause; Down, Down syndrome; ∼, not; CL, confidence limits.

## Discussion

### Risk of death

Mothers from all case groups had an increased risk of death during the study period. When we adjusted for maternal age at the index birth and SES, all hazard ratios were reduced but remained elevated, indicating that all case groups had an increased risk of death independent of their age at the index birth and SES.

Significant hazard ratios for death from any cause were found in mothers of children with intellectual disability of known cause (not Down syndrome), mothers of children with mild intellectual disability and mothers of children with ASD and intellectual disability. This suggests that these mothers were the most vulnerable to death of all the case groups during the study period.

The higher risk of death in the mothers of children with intellectual disability of known cause (not Down syndrome) may be due to the fact that some of these mothers have a genetic disorder, such as neurofibromatosis or Fragile X syndrome which was inherited by their child. [21] Associated comorbidities such as arterial, vascular and malignant neoplasms with neurofibromatosis, [22], [23] and anxiety, social phobia, and depression in pre-mutation carriers of Fragile X, may have been a contributing factor to the early death of these mothers. [24]

Children with mild intellectual disability may have an undiagnosed, inherited cause for their disability. In mothers of such children, researchers in US found various associated medical conditions such as hypertension, diabetes and thyroid disease. [25] These may have contributed to their mothers’ early deaths. Furthermore, we previously found that mothers of children with mild intellectual disability had an increased risk of epilepsy and asthma which may have also affected their mortality. [21] The higher mortality of mothers of children with ASD might be mediated by their higher risk of hospitalisation for a psychiatric disorder [26] or their increased stress. [27] Some maternal conditions such as diabetes and epilepsy also increase the risk of ASD in subsequent offspring. For example, women with diabetes were found to have nearly three times the risk, and women with epilepsy, around four times the risk of a subsequent child with ASD. [21] The higher risk of death in these mothers could relate to the increased prevalence of these conditions. Both smoking [28] and obesity [29] increase the mortality risk and could be mediators of the increased risks we identified in mothers of children with intellectual disability and ASD. We know that increased smoking is associated with higher levels of stress [30] and obesity with less exercise. [31] Mothers of children with ID or ASD have been shown to experience greater stress [32], [33]and one might expect that these time-poor women [34] also exercise less.

Researchers report that mothers of children with Down syndrome have less stress and increased subjective well-being than mothers of children with other forms of intellectual disability or ASD. [6], [35], [36] Furthermore, mothers of children with Down syndrome would not have health issues which are genetically related to their children's disability. Consistent with these factors, our results indicated that these mothers had the lowest risk of death during the study period of all case groups.

### Effect of a psychiatric disorder

Psychiatric disorders were more prevalent in all case groups than in the comparison group. The increased prevalence ranged from 18% higher in mothers of children with Down syndrome to about 50% higher in mothers of children with ASD and intellectual disability to more than double in mothers of children with mild ID (Table 1). Compared to mothers with no psychiatric disorder and no child with intellectual disability or ASD, we found that the poorest survival was in mothers with both a psychiatric disorder and a child with intellectual disability or ASD. We also showed that having a psychiatric disorder had greater impact on mortality than having a child with intellectual disability or ASD.

### Cause of death

Research has identified that cancer and stress are positively correlated [37] and hence the higher risk of death from cancer might be mediated through the higher levels of stress experienced by these mothers. However, there is also the possibility that these mothers have higher mortality from cancer but not a higher incidence. This could be a result of lower levels of self-care in these women resulting in reduced participation in cancer screenings for breast, cervical and bowel cancer. This might result from the increased time constraints they face in the care of their children with intellectual disability or ASD.

As with cancer, the increased risk of death due to cardiovascular diseases in mothers of children with intellectual disability or ASD might be caused by elevated stress levels compared to mothers of children without these disabilities since stress is also associated with an increased risk of cardiovascular disease. [38] Other contributing factors might be that women with psychiatric disorders have lower levels of self-care, including higher levels of smoking, [39] a risk factor for cardiovascular disease. [40] Furthermore, research has documented increased rates of this disease in people with a psychiatric illness. [10]

The category, *Misadventure* includes all causes of death in our data-set related to homicide, suicide or accident. One might hypothesize that mothers of children with intellectual disability or ASD are more vulnerable to misadventure because, due to the care of their child with a disability, they have more challenges in their everyday lives, [41] more depression, [42] and less sleep, [43], [44], known risk factors for accidents [45] and suicide. [46]

### Strengths and weaknesses

Western Australia's intellectual disability database, IDEA, made our study possible. Its main strength is the utilisation of data from a complete cohort with linkage of all relevant information. Further, our study investigates maternal death objectively and does not rely on the recall of family members for dates or causes of death. These two factors reduce bias and enhance the accuracy of our results. One weakness is the smaller numbers of children with ASD that severely limited analyses for this group in most areas. Another is that the categorisation of intellectual disability may be incomplete. For example, some children may have had their condition diagnosed subsequent to registration with the database. This would result in their mothers being wrongly allocated to the ‘Intellectual disability of unknown cause’ case groups instead of the ‘Intellectual disability of known cause (not Down syndrome)’ case group. A final weakness is that our comparison group would have included a small number of mothers of children with other disabilities such as blindness and cystic fibrosis. All we know of this group is the fact that their children have neither intellectual disability nor ASD. This fact would have attenuated our results.

### Summary

All maternal case groups had increased risk of death during the study period. Mothers with no psychiatric disorder and a child with intellectual disability or ASD were one and a half times as likely to die while mothers with a psychiatric disorder and no child with intellectual disability or ASD were more than four times as likely to die as mothers with no psychiatric disorder or no child with intellectual disability or ASD. This suggests that mothers having a psychiatric disorder has more impact on mortality than her having a child with intellectual disability or ASD. Cancer, cardio-vascular disease and misadventure were the three primary causes of death in case mothers. Mothers of children with either intellectual disability or ASD were between 35% and 40% more likely to die of cancer during the study period than mothers of children without these disabilities. They were also two and a half times more likely to die from cardio-vascular disease and nearly twice as likely to die as a result of misadventure as comparison mothers. We hypothesize that these increased hazards may be related to the increased stress of raising a child with these disabilities.

### Implications for the future

Apart from the *Cancer* category, small numbers in the case groups of mothers of children with ASD prohibited any analyses. We know of no other intellectual disability data-base. However, pooling our data with corresponding data from ASD registries from elsewhere might enable a greater understanding of factors increasing the mortality rates in mothers of children with ASD. In this way, informed services and preventions might be developed with the aim of improving the health and survival of these women.

## Supporting Information

S1 Table **Cause of death code in mothers by diagnostic category.**
(DOCX)

## References

[1] Norlin D, Broberg M (2013) Parents of children with and without intellectual disability: couple relationship and individual well-being. Journal of Intellectual Disability Research 57:552–566. 22533701 10.1111/j.1365-2788.2012.01564.x

[2] Caldwell J (2008) Health and access to health care of female family caregivers of adults with developmental disabilities. Journal of Disability Policy Studies 19:68–79.

[3] Sawyer M, Bittman M, La Greca A, Crettenden A, Harchak T, et al (2009) Time demands of caring for children with autism: What are the implications for maternal mental health? Journal of Autism and Developmental Disorders 40:620–628. 10.1007/s10803-009-0912-3 19949845

[4] Rizk S, Pizur-Barnekow K, Darragh A (2011) Leisure and social participation and health-related quality of life in caregivers of children with autism. Occupation, Participation and Health 31:164–171.

[5] Watt M, Wagner S (2012) Parenting a child with autism spectrum disorder: parental work context. Community, Work and Family 16:1–19.

[6] Abbeduto L, Seltzer M, Shattuck P, Krauss M, Orsmond G, et al (2004) Psychological well-being and coping in mothers of youths with autism, Down syndrome, or Fragile X syndrome. American Journal on Mental Retardation 109:237–254. 15072518 10.1352/0895-8017(2004)109<237:PWACIM>2.0.CO;2

[7] Dumas J, Wolf L, Fisman S, Culligan A (1991) Parenting stress, child behavior problems, and dysphoria in parents of children with autism, Down syndrome, behavior disorders, and normal development. Exceptionality 2:97–110.

[8] Sanders J, Morgan S (1997) Family stress and adjustment as perceived by parents of children with autism or Down syndrome: implications for intervention. Child and Family Behavior Therapy 19:15–32.

[9] Daniels J, Forssen U, Hultman C, Cnattingius S, Savitz D, Feychting M, et al (2008) Parental psychiatric disorders associated with autism spectrum disorders in the offspring. Pediatrics. 121(5):1357–62. 10.1542/peds.2007-2296 18450879

[10] Lawrence D, Kisely S, Pais J (2010) The epidemiology of excess mortality in people with mental illness. Canadian Journal of Psychiatry 55:752–760. 21172095 10.1177/070674371005501202

[11] Centers for Disease Control and Prevention (2012) Prevalence of autism spectrum disorders: Autism and Developmental Disabilities Monitoring Network, 14 Sites, United States, 2008. Morbidity and Mortality Weekly Report Surveillance Summaries 61. 22456193

[12] Bigby C, Fyffe C (2006) Tensions between institutional closure and deinstitutionalisation: What can be learned from Victoria's institutional redevelopment? Disability and Society 21:567–581.

[13] Petterson B, Leonard H, Bourke J, Sanders R, Chalmers R, et al (2005) IDEA (Intellectual Disability Exploring Answers): a population-based database for intellectual disability in Western Australia. Annals of Human Biology 32:237–243. 16096222 10.1080/03014460500075035

[14] Department of Health WA (2011) What we collect and manage.

[15] Woodward M (2005) Epidemiology: study design and analysis; Carlin B, Chatfield C, Tanner M, Zidek J, editors. Boca Raton, US: Chapman and Hall. 849 p.

[16] Institute for Digital Research and Education (2014) Supplemental notes to Applied Survival Analysis. Los Angeles: University of California, Los Angeles.

[17] Leonard H, Glasson E, Nassar N, Whitehouse A, Bebbington A, et al (2011) Autism and intellectual disability are differentially related to sociodemographic background at birth. PLoS ONE 6:e17875. 21479223 10.1371/journal.pone.0017875 PMC3068153

[18] Smith J (1999) Healthy bodies and thick wallets: the dual relation between health and economic status. Journal of Economic Perspectives 13:144–166. 15179962 PMC3697076

[19] Australian Bureau of Statistics (2012) Socio-economic indexes for areas: introduction, use and future directions. Canberra.

[20] Centers for Disease Control and Prevention (2002) ICD-10 Cause-of-Death Lists for Tabulating Mortality Statistics. US.

[21] Leonard H, de Klerk N, Bourke J, Bower C (2006) Maternal health in pregnancy and intellectual disability in the offspring: a population-based study. Annals of Epidemiology 16:448–454. 16182562 10.1016/j.annepidem.2005.05.002

[22] Finsterer J, Stollberger C, Stubenberger E. Tschakoschian (2013) Lymphangiopathy in neurofibromatosis 1 manifesting with chylothorax, pericardial effusion, and leg edema. International Journal of General Medicine 6:743–746. 24043952 10.2147/IJGM.S45825 PMC3772692

[23] Rasmussen S, Yang Q, Friedman J (2001) Mortality in neurofibromatosis 1: an analysis using US death certificates. American Journal of Human Genetics 68:1110–1118. 11283797 10.1086/320121 PMC1226092

[24] Hagerman R (2006) Lessons from Fragile X regarding neurobiology, autism, and neurodegeneration. Journal of Developmental and Behavioral Pediatrics 27:63–74. 16511373 10.1097/00004703-200602000-00012

[25] Yeargin-Allsopp M, Murphy C, Cordero J, Decouffle P, Hollowell J (1997) Reported biomedical causes and associated medical conditions for mental retardation among 10 year old children, metropolitan Atlanta, 1985 to 1987. Developmental Medicine and Child Neurology 39:142–149. 9112961 10.1111/j.1469-8749.1997.tb07401.x

[26] Daniels J, Forssen U, Hultman C, Cnattingius S, Savitz D, et al (2008) Parental psychiatric disorders associated with autism spectrum disorders in the offspring. Pediatrics 121:1357–1362. 10.1542/peds.2007-2296 18450879

[27] Bourke-Taylor H, Howie L, Law M, Pallant J (2012) Self-reported mental health of mothers with a school-aged child with a disability in Victoria: A mixed method study. Journal of Paediatrics and Child Health 48:153–159. 21470330 10.1111/j.1440-1754.2011.02060.x

[28] Bjartveit K, Tverdal A (2005) Health consequences of smoking 1–4 cigarettes per day. Tobacco Control 14:315–320. 16183982 10.1136/tc.2005.011932 PMC1748107

[29] Calle E, Thun M, Petrelli J, Rodriguez C, Heath C (1999) Body-Mass Index and mortality in a prospective cohort of U.S. adults. New England Journal of Medicine 341:1097–1105. 10511607 10.1056/NEJM199910073411501

[30] Kouvonen A, Kivimäki M, Virtanen M, Pentti J, Vahtera J (2005) Work stress, smoking status, and smoking intensity: an observational study of 46 190 employees. Journal of Epidemiology and Community Health 59:63–69. 15598729 10.1136/jech.2004.019752 PMC1763376

[31] Thiel C, Vogt L, Claußnitzer G. Banzer W Energy cost of youth obesity exercise modes. International Journal of Sports Medicine. 32:142–146. 21110288 10.1055/s-0030-1268436

[32] Baker B, McIntyre L, Blacher J, Crnic K, Edelbrock C, et al (2003) Pre-school children with and without developmental delay: behaviour problems and parenting stress over time. Journal of Intellectual Disabilty Research 47:217–230. 10.1046/j.1365-2788.2003.00484.x 12787154

[33] Baker-Ericzén M, Brookman-Frazee L, Stahmer A (2005) Stress levels and adaptability in parents of toddlers with and without autism spectrum disorders. Research and Practice for Persons with Severe Disabilities 30:194–204.

[34] Webster R, Majnemer A, Platt R, Shevell M (2008) Child health and parental stress in school-age children with a preschool diagnosis of developmental delay. Journal of Child Neurology 23:32–38. 18184941 10.1177/0883073807307977

[35] Hodapp R, Ly T, Fidler D, Ricci L (2001) Less stress, more rewarding: parenting children with Down syndrome. Parenting 1:317–337.

[36] Corrice A, Glidden L (2009) The Down syndrome advantage: fact or fiction? American Journal on Intellectual and Developmental Disabilities 114:254–268. 19642708 10.1352/1944-7558-114.4.254-268

[37] Chida Y, Hamer M, Wardle J, Steptoe A (2008) Do stress-related psychosocial factors contribute to cancer incidence and survival? Nature clinical practice Oncology 5:466–475. 10.1038/ncponc1134 18493231

[38] Black P, Garbutt L (2002) Stress, inflammation and cardiovascular disease. Journal of Psychosomatic Research 52:1–23. 11801260 10.1016/s0022-3999(01)00302-6

[39] Lawrence D, Mitrou F, Zubrick S (2009) Smoking and mental illness: results from population surveys in Australia and the United States. BMC Public Health 9:285. 19664203 10.1186/1471-2458-9-285 PMC2734850

[40] Anderson K, Odell P, Wilson P, Kannel W (1991) Cardiovascular disease risk profiles. American Heart Journal 121:293–298. 1985385 10.1016/0002-8703(91)90861-b

[41] Sen E, Yurtsever S (2007) Difficulties experienced by families with disabled children. Journal for Specialists in Pediatric Nursing 12:238–252. 17956372 10.1111/j.1744-6155.2007.00119.x

[42] Olsson M, Hwang C (2001) Depression in mothers and fathers of children with intellectual disability. Journal of Intellectual Disability Research 45:535–543. 11737541 10.1046/j.1365-2788.2001.00372.x

[43] Polimeni M, Richdale A, Francis A (2005) A survey of sleep problems in autism, Asperger's disorder and typically developing children. Journal of Intellectual Disability Research 49:260–268. 15816813 10.1111/j.1365-2788.2005.00642.x

[44] Richdale A, Andre F, Gavidia-Payne S, Cotton S (2000) Stress, behaviour, and sleep problems in children with an intellectual disability. Journal of Intellectual and Developmental Disability 25:147–161.

[45] Eoh H, Chung M, Kim S (2005) Electroencephalographic study of drowsiness in simulated driving with sleep deprivation. International Journal of Industrial Ergonomics 35:307–320.

[46] Takahashi Y (2001) Depression and suicide. Japan Medical Association Journal 44:359–363.

