# Supplementary material for: Early Mortality and Primary Causes of Death in Mothers of Children with Intellectual Disability or Autism Spectrum Disorder: A Retrospective Cohort Study
Source: PLoS One. 2014 Dec 23;9(12):e113430. doi: 10.1371/journal.pone.0113430 (PMC4275172; doi:10.1371/journal.pone.0113430)
Supplement: S1 Table — Cause of death code in mothers by diagnostic category. (DOCX) [file pone.0113430.s001.docx]

Table S1: Cause of death code in mothers by diagnostic category

| Category | ICD 9 code | ICD 10 code |
| --- | --- | --- |
| Infections or parasites | 1369, 3239, 3240, 3241, 3249, 3249, 3409 | A169, A391, A400, A403, A415, A419, A810, B169, B171, B182, B199, B200, B207, B227, B238, B24, B332, B449, B674, B942 |
| Cancer | 1419, 1479, 1489, 1510, 1519, 1529, 1531, 1533, 1535-1537, 1539-1542, 1550, 1551, 1570, 1571, 1579, 1580, 1590, 1623, 1625, 1629, 1706, 1715, 1719, 1723, 1726, 1727, 1729, 1742, 1744, 1748, 1749, 1809, 1819, 1830, 1889, 1890, 1910-1912, 1918, 1919, 1939, 1940, 1943, 1990, 1991, 2001, 2002, 2008, 2019, 2028, 2030, 2040, 2050, 2051 | C029, C07, C089, C099, C109, C140, C159, C161, C162, C169, C179, C180-C182, C184-C189, C19, C20, C210, C220, C221, C229, C23, C240, C241, C250, C251, C259, C260, C261, C269, C310, C329, C340, C341-C343, C349, C37, C402, C419, C433, C434, C435, C436, C437, C439, C442, C444, C449, C450, C459, C479, C480, C482, C494, C495, C499, C500, C501, C502, C503, C504, C505, C508, C509, C52, C530, C539, C541, C55, C56, C64, C66, C679, C693, C700, C710-C713, C718- C720, C725, C729, C73, C740, C741, C749, C750, C755, C759, C762, C763, C767, C786, C80, C812, C819, C822, C830, C833- C835, C837, C845, C851, C859, C900, C902, C910, C911, C919-C921, C924, C925, C959, C97 |
| Diabetes | 2500, 2773, 362 | E101, E104, E105, E109, E112, E115, E117, E119, E140- E142, E145, E149 |
| Cardiovascular diseases | 3941, 3949, 3969, 3970, 3989, 4049, 4109, 4140, 4149, 4151, 4160, 4209, 421, 4210, 422, 4229, 4240, 4249, 4251, 4254, 4275, 4279, 4280, 4281, 429, 4309, 4319, 4321, 4329, 4331, 4369, 4371, 4410, 4411, 4472, 4478, 7100 | I050, I059, I080, I089, I091, I099, I110, I119, I120,  I219, I250, I251, I255, I258, I259, I269, I270, I272,  I311, I330, I341, I350, I351, I359, I38, I409, I420 I426, I429, I456, I490, I499, I500, I509, I517, I519,  I600, I607--I610,I613, I619, I629, I630, I635, I639, I64, I670, I671, I678, I690, I710, I711, I714, I729, I779 |
| Respiratory diseases and pneumonia | 2770, 463, 4819, 4859, 4869, 4939, 4949, 4969, 5159, 5163, 5168 | J09, J110, J13, J152, J154, J159, J180, J181, J189, J42, J439, J440, J448, J449, J459, J46, J47, J690, J841, J849, J850, J851, J869, J969, J984, J988 |
| Digestive diseases | 389, 5301, 5314, 5350, 5532, 5679, 5698, 5712, 5713, 5715, 5718, 5733, 5770, 705 | K047, K221, K254, K296, K318, K37, K409, K439,  K519, K529, K550, K559, K564, K625, K631, K638, K650, K701, K703, K704, K709, K729, K746, K754, K767, K769, K802, K819, K829, K830, K85, K852, K859, K869, K922 |
| Kidney diseases | 5820, 5822, 5859, 5869, 5939 | N039, N179, N180, N189, N19 |
| Pregnancy complications | 6339, 6370, 6419, 6541, 6651, 6653, 6661, 6670, 6688, 6709, 6731, 6740, 6741 | O149, O721, O754, O85, O861 |
| Misadventure | 8051, 8100, 8120, 8121, 8139, 8147, 8150, 8151, 8159-8161, 8169, 8181, 8190, 8210, 8219, 8227, 8250, 8251, 8413, 8415, 8500, 8502, 8505, 8509, 8520, 8532, 8540, 8583, 8588, 8589, 8641, 8658, 8703, 8768, 8829, 8889, 8903, 8912, 8969, 9102, 9104, 9108, 9119, 9138, 9169, 9203, 9239, 9289, 9290, 9299, 9500, 9502- 9505, 9509, 9520, 9521, 9530, 9549, 9552, 9569, 9570, 9580, 9589, 9600, 9639, 9652, 9654, 9669, 9680, 9682, 9688, 9689, 9803, 9888 | E872, V030, V031, V041, V092, V174, V234, V235, V285, V406, V430, V435, V436, V445, V446, V470, V471, V475-V477, V479, V485, V486, V489, V494, V535, V536, V545, V800, V835, V846, V860, V878, V892, V950, W010, W100, W13, W19, W190, W192, W194, W220, W31, W34, W65, W650, W655, W67, W670, W69, W698, W708, W738, W74, W748, W750, W76, W760, W808, W809, W879, W990, X000, X009, X038, X040, X06, X09, X310, X399, X400, X41, X410, X412, X419, X42, X420, X429, X44, X440, X445, X448, X449, X45, X450, X468, X59, X599, X600, X604, X609, X61, X610, X615, X620, X63, X639, X64, X640, X644, X649, X67, X670, X674, X678, X679, X69, X70, X700, X704, X705, X708, X709, X71, X710, X718, X730, X740, X76, X780, X782, X80, X802, X804, X814, X815, X818, X82, X824, X84, X910, X94, X940, X944, X95, X99, X990, X994, X998, X999, Y00, Y000, Y008, Y034, Y04, Y040, Y069, Y08, Y09, Y090, Y098, Y099, Y140, Y148, Y158, Y190, Y208, Y260, Y31, Y324, Y330, Y340, Y348, Y450, Y600, Y830, Y836, Y848, Y850, Y86 |
| Genetic and congenital disorders | 3310, 3318, 3352, 7424, 7469, 7472, 7595, 7598 | Q070, Q209, Q211, Q213, Q218, Q249, Q251, Q254,  Q273, Q279 |
| Mental disorders | 2918, 2940, 3039, 3040, 3041, 3047, 3048, 3049, 3050, 3071 | NO ICD 10 codes |
| Other causes | 2127, 2270, 2609, 2780, 2790, 2791, 3453, 3459, 4511, 6144, 7101, 7109, 7140, 785, 7982, 7989, 799, 7998, 7999 | D350, D373, D379, D383, D430, D432, D469, D593, D619, D649, D65, D688, D689, D693, D696, D735, D849, D869, E055, E668, E669, E752, E780, E785, E840, E849, E854, E859, F03, F100- F102, F109, F112, F162, F191, F192, F199, F329, G060, G061, G10, G122, G239, G319, G35, G409, G419, G713, G903, G931, G934, G939, G961, I802, I81, I828, L031, L930, M311, M313, M321, M329, M331, M348, M349, N110, N12, N139, N390, N719, N800, N832, Q282, R568, R98, R99, W790, W795, X458 |
